# Somatic symptom disorder in patients with myocardial bridge: cross-sectional study in China

**DOI:** 10.1192/bjo.2024.851

**Published:** 2025-03-24

**Authors:** Zhengyu Tao, Yani Wu, Yongxia Qiao, Zi Wang, Yezi Chai, Qizhen Wu, Yinan Wang, Xinning Guo, Chen Wu, Jialiang Mao, Meng Jiang, Jun Pu

**Affiliations:** Division of Cardiology, State Key Laboratory of Systems Medicine for Cancer, Renji Hospital, School of Medicine, Shanghai Jiao Tong University, Shanghai, China; School of Public Health, School of Medicine, Shanghai Jiao Tong University, Shanghai, China

**Keywords:** Somatic symptom disorder, epidemiology, myocardial bridge, anxiety disorders, depressive disorders

## Abstract

**Background:**

Myocardial bridge contributes to chest pain, often accompanied by non-specific complaints.

**Aims:**

Our study aims to determine somatic symptom disorder (SSD) prevalence in patients with myocardial bridge, investigating associated clinical and psychological features.

**Method:**

In this prospective cross-sectional study, we enrolled 1357 participants (337 with and 1020 without myocardial bridge) from Shanghai Renji Hospital. The Somatic Symptom Scale-China questionnaire was used to assess SSD. Depressive and anxiety disorders were assessed by the Patient Health Questionnaire-9 (PHQ-9) and Generalised Anxiety Disorder-7 (GAD-7).

**Results:**

The prevalence of SSD in the myocardial bridge group was 63.2%, higher than the group without myocardial bridge (53.8%). Patients with myocardial bridge were at an increased risk of SSD (odds ratio 1.362, 95% CI 1.026–1.809; *P* = 0.033). There were no differences in the mean PHQ-9 scores (3.2 ± 3.4 *v*. 3.2 ± 4.1; *P* = 0.751) or GAD-7 scores (2.5 ± 3.0 *v*. 2.3 ± 3.7; *P* = 0.143) between the two groups. Among patients with myocardial bridge, gender was the only independent risk factor for SSD. Women were 3.119 times more likely to experience SSD compared with men (95% CI 1.537–6.329; *P* = 0.002).

**Conclusions:**

Our findings emphasise the high prevalence and severity of SSD among patients with myocardial bridge. The screening for SSD should be of particular concern, especially among female patients.

Myocardial bridge, a congenital coronary anomaly,^1^ is a significant contributor to chest pain and cannot be ignored.^2^ The prevalence is significantly high in autopsy studies, reaching 33–42%, compared with the 19–22% identified by coronary computed tomography angiography.^3^ The chest discomfort caused by myocardial bridge is partially attributed to the presence of myocardium overlaying a major epicardial coronary artery. The artery is compressed during myocardial contraction in each systole, resulting in symptoms such as chest pain, shortness of breath and palpitation.

## Somatic symptom disorder in medical settings

Somatic symptom disorder (SSD) is one of the most prevalent psychological issues in many medical settings,^4^ with prevalence ranging from 5.8 to 52.9%.^5^ According to the DSM-5,^6^ SSD is characterised by distressing somatic symptoms that significantly disrupt daily functioning. Individuals with SSD often experience excessive and disproportionate thoughts, feelings and behaviours related to their physical symptoms. Numerous studies have highlighted the high prevalence and substantial burden of SSD, which markedly impairs patients’ quality of life.^5–8^ Among patients with cardiovascular disease, particularly those with coronary heart disease (CHD), research by Kohlmann et al indicated that somatic symptoms are both prevalent and burdensome, with a strong link between cardiac health and symptom severity.^9^ Notably, patients with CHD reported somatic symptoms extending beyond the cardiovascular system, such as sleep disturbances and pain in the arms, legs or joints.^9^ These findings underscore the close relationship between cardiovascular disease and SSD. Therefore, we hypothesise that SSD may also be associated with myocardial bridge, as patients with myocardial bridge frequently report severe chest pain and other related symptoms. Given the uncertain clinical relevance of SSD in patients with myocardial bridge, further research is urgently needed for optimal assessment and effective management of their diverse complaints and mental health issues.

## The current study

We conducted a prospective cross-sectional study to assess the physical and psychological characteristics of patients with myocardial bridge in Renji Hospital, Shanghai, China. To screen for SSD, we used the Somatic Symptom Scale-China (SSS-CN), a questionnaire we previously developed.^10^ Unlike the Somatic Symptom Disorder - B Criteria Scale, which primarily focuses on psychological feelings and views somatic discomfort as a general concept affecting the individual, the SSS-CN is a comprehensive scale that assesses a combination of psychological, behavioural and somatic symptoms based on the DSM-5 criteria.^10^ It is also designed to evaluate both the presence and severity of symptoms. The reliability and validity of this instrument have been validated,^10^ and it has been applied it in a large-scale, cross-sectional study in China.^11,12^ The purpose of our study was threefold: (a) to ascertain the prevalence of SSD, depressive disorders and anxiety disorders in patients with myocardial bridge; (b) to elucidate the relationship between clinical features and the occurrence of SSD; and (c) to investigate the risk factors for SSD in patients with myocardial bridge.

## Method

### Study participants

Study participants were drawn from the database of the EARLY-MYO-SSS-CN (EARLY assessment of somatic symptom disorder in patients with MYOcardial bridge by SSS-CN) registry, a single-centre registry of patients with suspected coronary artery disease (CAD) who underwent cardiac angiography and undertook the SSS-CN self-report (ClinicalTrials.gov identifier NCT04664387). Participants were enrolled between 30 October 2016 and 8 May 2020, from the Cardiology Department of Shanghai Renji Hospital in China.

Inclusion criteria were as follows: (a) in-patients of the Cardiology Department who were aged 18 years or older, with chest discomfort suggestive of CAD; (b) completion of the SSS-CN, Patient Health Questionnaire-9 (PHQ-9) and Generalised Anxiety Disorder-7 (GAD-7) questionnaires; (c) were undergoing cardiac angiography and (d) voluntary participation with written consent. Exclusion criteria were as follows: (a) patients who were in a health emergency or were unconsciousness; (b) patients who lacked self-assessment abilities or refused participation; (c) participants who completed the questionnaires after the cardiac angiography; (d) patients who had previously been diagnosed with mental disorders, developmental disorders or dementia; (e) patients who currently took antianxiety or antidepression agents; (f) patients who had been diagnosed with heart diseases other than CAD; and (g) any missing data within questionnaire items or more than one item missing from sociodemographic information.

The authors assert that all procedures contributing to this work comply with the ethical standards of the relevant national and institutional committees on human experimentation and with the Helsinki Declaration of 1975, as revised in 2008. Ethical approval was provided by the Renji Hospital Human Research Ethics Committee, under approval number 2015016 (ClinicalTrials.gov identifier NCT04664387).

### Definition of myocardial bridge

Myocardial bridge is the term for the muscle overlying the intramyocardial segment of the epicardial coronary artery.^1^ The diagnosis of myocardial bridge is based on the performance of lumen contraction on the coronary angiography. The diagnosis criterion is that the segment of the coronary artery presents transient systolic stenosis in multiple projection angles and complete or partial decompression during the diastolic period.^2,13^ The presence of myocardial bridge was used as a criterion to classify participants.

### Description of the SSS-CN and assessment of SSD

The SSS-CN questionnaire, derived from DSM-5 criteria, assesses SSD with 20 self-administered items across four dimensions: physical disorder, anxiety disorder, depression disorder and anxiety–depression disorder. We validated its reliability and validity in previous studies.^10,11,14^ Briefly, the test–retest reliability of the scale was 0.96, and the Cronbach’s α coefficient was 0.89. The correlation coefficients between each dimension and the total ranged from 0.76 to 0.88, and the correlation coefficients within dimensions ranged from 0.56 to 0.70. A cut-off value of 30 yielded an optimal sensitivity of 0.96 and a specificity of 0.86.^14^ Scores of 20–29, 30–39, 40–59 and 60–80 represent normal, mild, moderate, moderate and severe SSD, respectively (see Supplementary Fig. 1 available at https://doi.org/10.1192/bjo.2024.851).

### Assessment of depressive and anxiety disorders

All participants were assessed for depressive and anxiety disorders according to the self-administered PHQ-9 and GAD-7, which evaluate symptom frequencies over the past 2 weeks, ranging from 0 (‘not at all’) to 3 (‘nearly every day’). PHQ-9 cut-offs are as follows: 5–9 (mild depressive symptoms), 10–14 (moderate), 15–19 (moderately severe) and ≥20 (severe).^15^ GAD-7 cut-offs are as follows: 5–9 (mild anxiety symptoms), 10–14 (moderate) and ≥15 (severe).^16^

### Data collection and management

The study was conducted by trained surveyors. Participants filled out the scales in a separate room, and the research assistant would help them understand the question. Sociodemographic data and clinical characteristics of the patients, such as gender, age, history of smoking, history of hypertension and diabetes, were collected at admission. Then, patients underwent biochemical blood tests, electrocardiography, echocardiography and coronary angiography, which were performed by technicians and interventional cardiologists who were blind to the patient’s response to the above three scales. An independent diagnosis about the degree of coronary stenosis and myocardial bridge were at the discretion of the interventional cardiologist.

Data were entered into the EpiData 3.1 (EpiData for Windows; EpiData Association, Odense, Denmark; https://epidata.dk/) database, using double-entry data. A unique identifier number was assigned to each participant. Proper categorisation and coding of data were performed during the data-cleaning phases. Database access was password-protected, and access was restricted to key team personnel.

### Statistical analyses

Continuous variables were presented as mean ± s.d. Categorical variables were displayed as the frequency and percentage. Student’s *t*-test, one-way analysis of variance and Mann–Whitney *U*-test were used for the comparison of continuous data between groups, and comparison of the categorical data was performed with the chi-squared test. Univariate analyses and binary logistic regression models were used to assess the effects of sociodemographic factors and clinical characteristics on SSD in the patients. Odds ratios, 95% confidence intervals and significance values (*P*-values) were reported. A significance level was set at *P* < 0.05 (two-tailed). The baseline variables were used in the univariate analysis to find out the significant predictors of the prevalence of SSD. All statistical analyses were performed with SPSS version 22 (IBM SPSS Statistics for Windows, Version 22.0, Armonk, New York, USA; https://www.ibm.com/support/pages/spss-statistics-220-available-download) and RStudio interface (RStudio Inc, Boston, Massachusetts, USA; https://posit.co/download/rstudio-desktop/).

## Results

### Study population

From 30 October 2016 to 8 May 2020, 1989 potential participants underwent screening, of which 1357 individuals met the inclusion criteria and were subsequently enrolled (see Supplementary Fig. 2). Among them, 337 patients (24.8%) were diagnosed with myocardial bridge. Sociodemographic and clinical characteristics are described in Supplementary Table 1. Notably, the patients in the myocardial bridge group were younger (*P* < 0.001), predominantly female (44.2 *v*. 35.2%), presented with fewer established cardiovascular risk factors such as smoking, hypertension and diabetes mellitus (*P* < 0.05), and had a lower prevalence of myocardial infarction (*P* < 0.05). In the non-myocardial bridge group, more patients underwent percutaneous coronary intervention (PCI) (49.2 *v*. 32.3%; *P* < 0.001). Moreover, the extent of concomitant coronary stenosis was observed to be less severe in the myocardial bridge group (43.6 *v*. 61.6%; *P* < 0.001). In terms of medication, it is noteworthy that aspirin and calcium channel blockers were more frequently used in the myocardial bridge group (*P* < 0.001).

### Mental health status

**Fig. 1:**
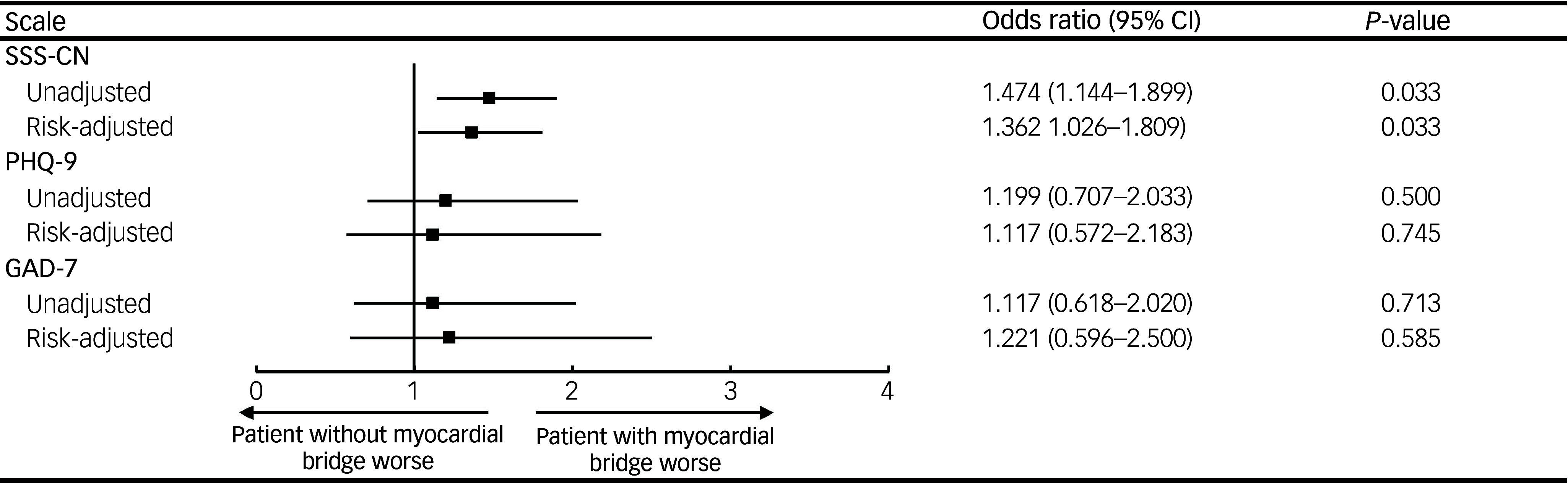
Odds ratios of somatic symptom disorder, depressive disorder and anxiety disorder between myocardial bridge and non-myocardial bridge groups. The non-myocardial bridge group was set as the reference category (reference: 1). Binary logistic regression analyses were performed to estimate the odds ratios, adjusting for gender, age, smoking, hypertension, diabetes mellitus, myocardial infarction and percutaneous coronary intervention.

The myocardial bridge group demonstrated a significantly higher SSS-CN score compared with the non-myocardial bridge group (33.7 ± 8.3 *v*. 31.7 ± 7.8; *P* < 0.001), with elevated somatic (17.2 ± 4.3 *v*. 16.3 ± 4.2), anxiety (5.9 ± 1.9 *v*. 5.5 ± 1.7), depression (6.6 ± 2.3 *v*. 6.2 ± 2.1), and anxiety and depression scores (4.0 ± 1.4 *v*. 3.6 ± 1.3) (*P* < 0.05). The prevalence of SSD in the myocardial bridge group was 63.2%, surpassing that in the non-myocardial bridge group (53.8%; *P* = 0.003), with 39.8% being mild, 23.1% being moderate and 0.3% being severe. As depicted in Fig. 1, binary logistic regression analyses revealed that the patients with myocardial bridge have an increased risk of SSD after adjustment for confounding factors (odds ratio 1.362, 95% CI 1.026–1.809; *P* = 0.033). Furthermore, the mean number of reported somatic symptoms differed significantly between two groups (10.0 ± 4.9 *v*. 8.7 ± 4.8; *P* < 0.001). Interestingly, there were no significant differences in the mean PHQ-9 scores (3.2 ± 3.4 *v*. 3.2 ± 4.1; *P* = 0.751) or GAD-7 scores (2.5 ± 3.0 *v*. 2.3 ± 3.7; *P* = 0.143) between either group, and the proportion of patients with anxiety or depressive disorders did not vary significantly (Supplementary Table 2).

### Association of sociodemographic and clinical predictors with SSD

The investigation focused on identifying sociodemographic and clinical predictors associated with SSD. Parameters in Supplementary Table 1 were included in both univariate analyses and binary logistic regression models. Results revealed that female gender and the presence of myocardial bridge were the two independent risk factors for SSD occurrence (Supplementary Table 3). Women were 3.104 times more likely to have SSD compared with men (95% CI 2.353–4.093; *P* < 0.001), and patients with myocardial bridge were 1.362 times more likely to have SSD compared with those without myocardial bridge (95% CI 1.026–1.809; *P* = 0.033). It is worth noting that although univariate analyses initially indicated associations between SSD and factors like smoking, myocardial infarction, PCI and coronary stenosis, these associations lost their significance after adjusting for potential confounding variables.

Given the high prevalence of SSD in the myocardial bridge group, a more comprehensive investigation considered the degree of systolic compression and myocardial bridge type (Table 1). In multivariate analyses, gender remained the only independent risk factor for SSD in patients with myocardial bridge. Women were 3.119 times more likely to experience SSD compared with men (95% CI 1.537–6.329; *P* = 0.002).

**Table 1:** Univariate analyses and binary logistic regression models of risk factors of somatic symptom disorder among patients with myocardial bridge

	Unadjusted odds ratio (95% CI)	*P*-value	Adjusted odds ratio (95% CI)	*P*-value
Age	1.012 (0.990–1.033)	0.289	0.985 (0.953–1.017)	0.356
Female	4.148 (2.523–6.817)	<0.001	3.119 (1.537–6.329)	0.002
Smoking (yes)	0.438 (0.234–0.819)	0.01	0.658 (0.259–1.675)	0.381
Hypertension	1.294 (0.828–2.022)	0.258	1.090 (0.554–2.143)	0.803
Diabetes mellitus	0.710 (0.415–1.217)	0.213	0.646 (0.279–1.495)	0.308
Prior myocardial infarction	0.959 (0.462–1.991)	0.910	0.546 (0.135–2.214)	0.397
Prior PCI	0.710 (0.444–1.136)	0.154	1.252 (0.534–2.933)	0.605
Degree of systolic compression	1.000 (0.982–1.017)	0.959	1.004 (0.985–1.024)	0.659
Complex myocardial bridge^a^	0.628 (0.392–1.007)	0.053	0.864 (0.413–1.809)	0.699

PCI, percutaneous coronary intervention.

a Complex myocardial bridge was defined as patients with more than 30% coronary stenosis who were also detected with myocardial bridge.

### Characteristics of SSD in patients with myocardial bridge

Supplementary Table 4 shows the detection rate of 20 SSS-CN items among patients with myocardial bridge. The three most frequently reported somatic symptoms were chest pain (86.6%), feeling tired (66.8%) and reduced attention (66.8%).

**Fig. 2:**
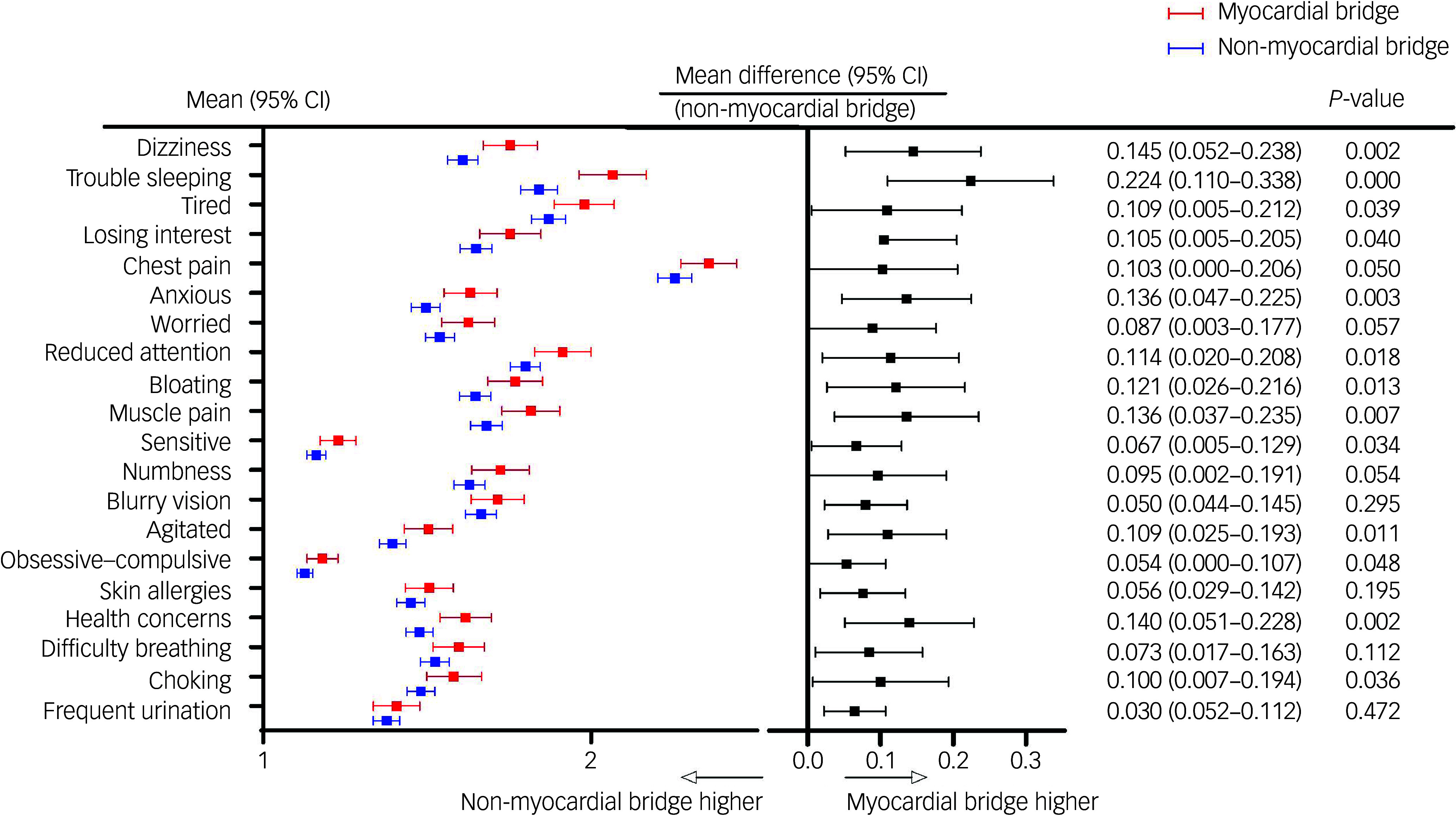
Comparison of mean values of 20 items between patients with or without myocardial bridge, using independent two-sample *t*-tests. Each item demonstrated higher scores among patients with myocardial bridge. GAD-7, Generalised Anxiety Disorder-7; PHQ-9, Patient Health Questionnaire-9; SSS-CN, Somatic Symptom Scale-China.

The analysis of the 20 SSS-CN items between patients with and without myocardial bridge (Fig. 2) used independent two-sample *t*-tests for each item. Consistently higher scores were observed in patients with myocardial bridge. Specifically, 13 items exhibited significantly higher scores: dizziness (*P* = 0.002), trouble sleeping (*P* < 0.001), feeling tired (*P* = 0.039), losing interest (*P* = 0.040), anxious (*P* = 0.003), reduced attention (*P* = 0.018), bloating (*P* = 0.013), muscle pain (*P* = 0.007), sentimental (*P* = 0.034), easily agitated (*P* = 0.011), obsessive–compulsive (*P* = 0.048), health concerns (*P* = 0.002) and choking (*P* = 0.036). The most significant mean difference between patients with and without myocardial bridge was observed in the item ‘trouble sleeping’ (mean difference 0.224, 95% CI 0.110–0.338), followed by ‘health concerns’ (mean difference 0.140; 95% CI 0.051–0.228).

### Association between degree of systolic compression and SSS-CN score in patients with myocardial bridge

**Fig. 3:**
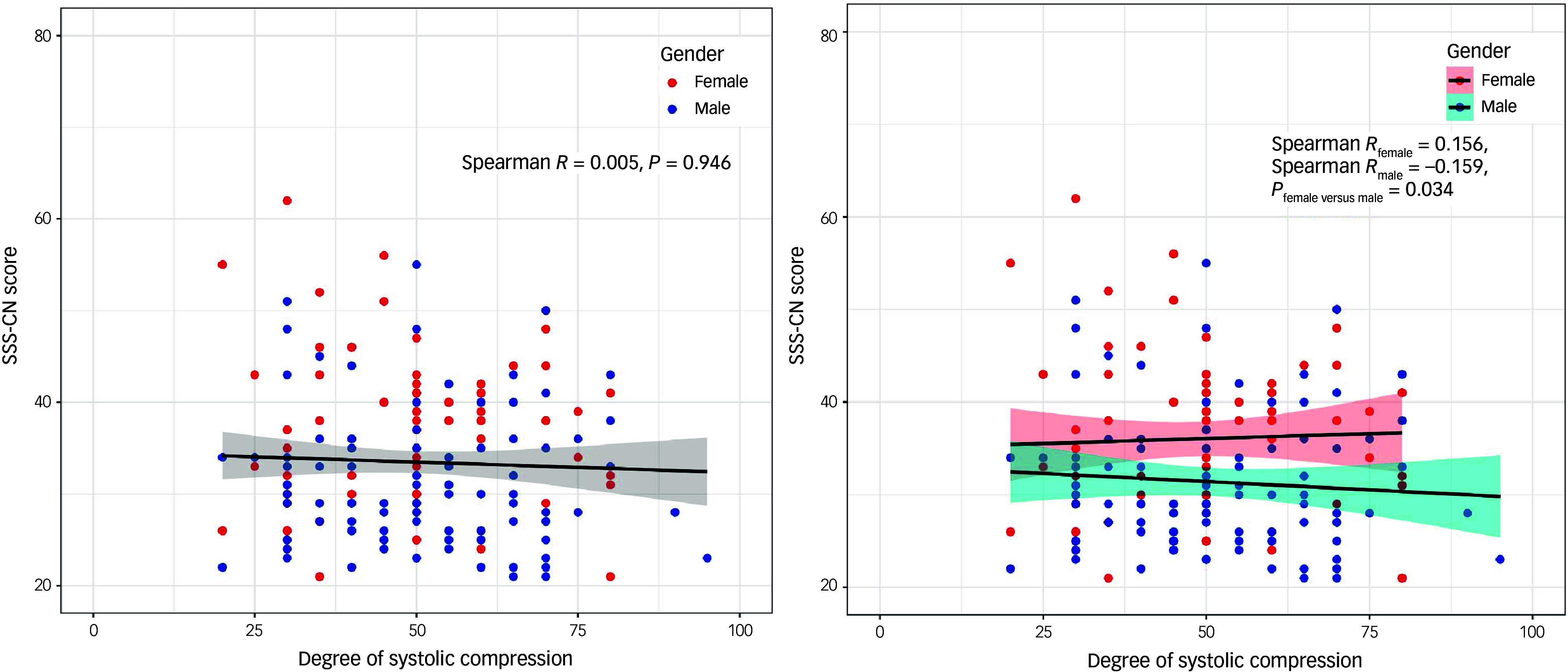
Association of degree of systolic compression and Somatic Symptom Scale-China (SSS-CN) score in patients with myocardial bridge. Analyses were conducted with Spearman correlation coefficients. Red dots represent women, blue dots represent men and the lines depict the correlation trends between the degree of systolic compression and SSS-CN score. The subfigure on the right provides a gender-based subgroup analysis to observe distinct correlation trends between different genders.

A total of 182 patients with myocardial bridge provided comprehensive data on the degree of systolic compression, facilitating an examination of the association between the degree of systolic compression and SSS-CN scores. Spearman correlation analysis (Fig. 3) showed a non-significant correlation (Spearman’s *r* = 0.005, *P* > 0.05). However, subgroup analysis by gender showed an interesting pattern. For women and men, there seemed to be opposite trends in their respective correlations, although both correlations remained statistically non-significant.

## Discussion

Myocardial bridge was first documented in the 15th century, with chest discomfort as the typical complaint. Our study was prompted by the persistence of unexplained physical discomfort in patients, even after medications such as beta-blockers or diltiazem were administered. To the best of our knowledge, this is the first prospective cross-sectional study focusing on the clinical characteristics of SSD in patients with myocardial bridge. Our research unveiled that the prevalence of SSD was notably higher in patients with myocardial bridge compared with those without myocardial bridge. However, there was no significant difference in the prevalence of depressive or anxiety disorders. Interestingly, we observed that the degree of systolic compression was not linked to a higher prevalence or greater severity of SSD, which was contrary to our initial hypothesis. Additionally, our findings revealed that among patients with myocardial bridge, female gender was independently associated with a higher prevalence of SSD.

### Higher prevalence of SSD among patients with myocardial bridge

Our results showed that 24.8% of patients initially suspected as having CAD were subsequently diagnosed with myocardial bridge, which aligned with previous findings reporting a prevalence of 17.9–40%.^1,2,17^ Among patients with myocardial bridge, 63.2% were also diagnosed with SSD, which was significantly higher than those without myocardial bridge (53.8%). Additionally, we discovered that patients with myocardial bridge tend to experience a higher frequency of non-cardiac somatic symptoms, such as dizziness, sleep disturbances, fatigue and reduced interest. This suggests that myocardial bridge is associated with various bodily, cognitive and emotional discomfort that extended beyond the cardiovascular system. These findings are consistent with a previous study focusing on ischemia with non-obstructive coronary artery disease, where 77.8% of patients presented with non-cardiac symptoms, further highlighting the close relationship between myocardial ischemia and mental health.^18^

The high prevalence of SSD and related symptoms has several adverse effects, both individually and socially. First, SSD may exacerbate physical diseases and potentially lead to a poorer prognosis. Previous studies reported that somatic symptoms were closely associated with autonomic nervous system dysfunction,^19–22^ such as reduced cardiac autonomic flexibility,^22^ suggesting that SSD may influence the normal physiological functions of the cardiovascular system. Many previous studies also revealed that somatic symptoms are prospectively associated with cardiac prognosis among patients with myocardial infarction,^23^ stable CHD,^24^ congestive heart failure^25^ or other cardiovascular diseases.^26^ Second, significant elevations were noted in nine depression and anxiety items on the SSS-CN, with sleep disturbances being particularly prominent, affecting 66.5% of patients with myocardial bridge. Previous studies revealed that sleep disturbance is associated with serious organic diseases,^27^ including myocardial infarction.^28^ This indicates that SSD might contribute to other physical conditions in patients with myocardial bridge, which should be examined in future research. Finally, from a socioeconomic perspective, myocardial bridge-related discomfort may drive patients to seek frequent medical consultations. This could lead to numerous, often unnecessary, laboratory examinations in the pursuit of even a small measure of relief, which unfortunately results in a wasteful consumption of valuable medical resources.

Considering the high prevalence of SSD among patients with myocardial bridge and its detrimental impacts, routine screening for SSD is crucial. When necessary, appropriate and timely interventions, such as antipsychotic medications and neurocognitive–behavioural therapy, should be considered to manage the condition effectively.

### Similar prevalence of depressive or anxiety disorder in patients with or without myocardial bridge

It is important to note that, in contrast to previous research linking depressive and anxiety disorders to chest pain or CHD,^29–31^ our study revealed no significant difference in the prevalence of depressive disorder (28.2 and 25.4%, respectively) or anxiety disorder (20.5 and 18.7%, respectively) between the myocardial bridge and non-myocardial bridge groups. This disparity may be related to the specific demographics of our study. Our participants were in-patients suspected of having CAD who underwent coronary angiography, as opposed to a community-based population. The majority of patients without myocardial bridge in our study were diagnosed with other forms of CAD, such as atherosclerotic heart disease. Moreover, patients currently taking antianxiety or antidepression agents were excluded, which may also contribute to an underestimation of the true prevalence of depression or anxiety in the study population. Our previous research involved community-based populations, where the prevalences of depressive and anxiety disorders were considerably lower, at 17.0 and 11.2%, respectively.^11^ Based on previous studies^29–31^ and our cross-sectional study of the community population, it is evident that patients with myocardial bridge and those with other heart conditions, irrespective of the presence of myocardial bridge, exhibit a clear trend of higher prevalence of depressive and anxiety disorders.

### Relationship between female gender and higher SSD prevalence

As illustrated in Table 1, female gender was independently associated with higher SSD prevalence among patients with myocardial bridge. Several potential underlying mechanisms can be considered. First, despite women having lower cardiovascular disease risk factors,^32^ previous studies have indicated that women are more likely than men to experience non-obstructive CAD.^33,34^ Another study found that women with acute coronary syndromes do have different symptoms at presentation than men.^35^ It is reasonable to speculate that female patients with myocardial bridge may exhibit more non-specific somatic symptoms, potentially contributing to the development of SSD. Second, female gender has long been recognised as a risk factor for various mental disorders, including depression^36^ and anxiety disorder.^37^ Several physiological mechanisms have been proposed, including the involvement of ventral hippocampus-nucleus accumbens neurons,^38^ which are influenced by testosterone and contribute to female susceptibility to stress. This could partially explain why female patients with myocardial bridge appear to be more vulnerable to SSD. Further research is needed to better understand this association and its underlying mechanisms.

### Strengths and limitations

This study is the first to investigate the clinical characteristics of SSD among patients with myocardial bridge. The relevant demographic and clinical features, and risk factors, of the patients diagnosed with SSD are emphasised. We believe that our findings could facilitate early detection and improve the optimal treatment of SSD in individuals with myocardial bridge.

There are also some limitations in our study. First, the current cross-sectional study only established correlations, and was insufficient to determine the causal factors underlying the development of SSD. Second, this study was based on self-reported measures rather than semi-structured interviews. Moreover, excluding patients who were currently taking antianxiety or antidepression agents may have at least partially contributed to the lack of difference in anxiety or depression between the two groups. However, it is important to note that even after excluding these patients, significant differences in SSD were still observed.

In conclusion, our findings demonstrate that patients with myocardial bridge have a higher prevalence of SSD than those without myocardial bridge. Trouble sleeping, tiredness and attention reduction emerge as the most prevalent non-specific symptoms. Female gender was identified as an extremely vulnerable subgroup, and it is imperative to prioritise the screening of these patients during their hospital presentation.

## Supporting information

Tao et al. supplementary material Tao et al. supplementary material

## Data Availability

The data used in this study are not publicly available due to their sensitive nature. Data sharing for some of the included data-sets may be possible depending on ethical approval. Further enquiries can be directed to the corresponding author, M.J.
