# Supplementary material for: Somatic symptom disorder in patients with myocardial bridge: cross-sectional study in China
Source: BJPsych Open. 2025 Mar 24;11(2):e67. doi: 10.1192/bjo.2024.851 (PMC12001947; doi:10.1192/bjo.2024.851)
Supplement: Tao et al. supplementary material [file S2056472424008512sup001.docx]

| **Table S1 Sociodemographic and Clinical Characteristics** | | | | |
| --- | --- | --- | --- | --- |
|  | **Total** | **Myocardial Bridge** | **Non-myocardial Bridge** | **P value**^a^ |
|  | **(n=1357)** | **(n=337)** | **(n=1020)** |  |
| **Age, mean±SD,** **y** | 65±10 | 64±11 | 66±10 | <0.001 |
| **Female, n(%)** | 506 (37.4) | 148 (44.2) | 358 (35.2) | 0.003 |
| **Cardiovascular risk factors** |  |  |  |  |
| **Smoke, n(%)** | 238 (18.8) | 47 (14.8) | 191 (20.1) | 0.038 |
| **Hypertension, n(%)** | 871 (64.7) | 189 (56.4) | 682 (67.4) | <0.001 |
| **Diabetes mellitus, n(%)** | 347 (25.8) | 69 (20.6) | 278 (27.6) | 0.012 |
| **Prior MI, n(%)** | 191 (14.2) | 34 (10.2) | 157 (15.5) | 0.018 |
| **Prior PCI, n(%)** | 604 (45.0) | 108 (32.3) | 496 (49.2) | <0.001 |
| **Extent of Coronary stenosis, mean±SD,%** | 57.1±37.0 | 43.6±37.0 | 61.6±35.9 | <0.001 |
| **β-Blocker, n(%)** | 123 (13.6) | 35 (13.6) | 88 (13.5) | 0.975 |
| **Aspirin, n(%)** | 219 (24.1) | 93 (36.2) | 126 (19.4) | <0.001 |
| **CCBs, n(%)** | 23 (2.5) | 15 (5.8) | 8/650(1.2) | <0.001 |
| SD: standard deviation, MI: myocardial infarction, PCI: percutaneous coronary intervention, SSD: somatic syndrome disorder; CCBs: calcium channel blockers  ^a^ Student's t-test was used for comparing continuous data between groups, and the chi-square test was used for comparing categorical data. | | | | |

| **Table S2. Mental Health Status in Patients with MB Compared with Patients without MB** | | | |  |
| --- | --- | --- | --- | --- |
|  | **Total** | **Myocardial Bridge** | **Non-myocardial Bridge** | **P value**^a^ |
|  | **(n=1357)** | **(n=337)** | **(n=1020)** |  |
| **SSS-CN score, mean±SD** | 32.2±8.0 | 33.7±8.3 | 31.7±7.8 | <0.001 |
| **Somatic cluster, mean±SD** | 16.5±4.2 | 17.2±4.3 | 16.3±4.2 | 0.001 |
| **Anxiety cluster, mean±SD** | 5.6±1.8 | 5.9±1.9 | 5.5±1.7 | <0.001 |
| **Depression cluster, mean±SD** | 6.3±2.1 | 6.6±2.3 | 6.2±2.1 | 0.014 |
| **Anxiety and depression cluster, mean±SD** | 3.7±1.4 | 4.0±1.4 | 3.6±1.3 | <0.001 |
| **Number of somatic symptom items reported, mean±SD** | 9.0±4.9 | 10.0±4.9 | 8.7±4.8 | <0.001 |
| **PHQ-9 score, mean±SD** | 3.2±4.0 | 3.2±3.4 | 3.2±4.1 | 0.751 |
| **GAD-7 score, mean±SD** | 2.4±3.6 | 2.5±3.0 | 2.3±3.7 | 0.143 |
| **SSD, n(%)** | 762 (56.2) | 213 (63.2) | 549 (53.8) | 0.003 |
| **Mild SSD** | 510 (37.6) | 134 (39.8) | 376 (36.9) | 0.009 |
| **Moderate SSD** | 247 (18.2) | 78 (23.1) | 169 (16.6) |  |
| **Severe SSD** | 5 (0.4) | 1 (0.3) | 4 (0.4) |  |
| **Anxiety Disorder, n(%)** | 88 (19.0) | 17 (20.5) | 71 (18.7) | 0.713 |
| **Depressive Disorder, n(%)** | 120 (26.0) | 24 (28.9) | 96 (25.3) | 0.500 |
|  |  |  |  |  |

^a^ Student's t-test was used for comparing continuous data between groups, and the chi-square test was used for comparing categorical data.

| **Table S3. Univariate Analyses and Binary Logistic Regression Models of Risk Factors of SSD in All Patients** | | | | |
| --- | --- | --- | --- | --- |
|  | **Unadjusted OR** | **P value** | **Adjusted OR** | **P value** |
|  | **(95% CI)** |  | **(95% CI)** |  |
| **Age** | 1.012 (1.001, 1.023) | 0.027 | 1.009 (0.997, 1.021) | 0.163 |
| **Female** | 3.444 (2.708, 4.381) | <0.001 | 3.104 (2.353, 4.093) | <0.001 |
| **Smoke** | 0.581 (0.437, 0.771) | <0.001 | 0.961 (0.705, 1.312) | 0.803 |
| **Hypertension** | 1.051 (0.840, 1.317) | 0.663 | 1.055 (0.820, 1.357) | 0.679 |
| **Diabetes mellitus** | 0.955 (0.747, 1.221) | 0.713 | 1.122 (0.851, 1.480) | 0.414 |
| **Prior MI** | 0.645 (0.474, 0.876) | 0.005 | 0.923 (0.647, 1.316) | 0.659 |
| **Prior PCI** | 0.638 (0.513, 0.793) | <0.001 | 0.853 (0.653, 1.116) | 0.247 |
| **Coronary stenosis** | 0.994 (0.991, 0.997) | <0.001 | 0.998 (0.995, 1.002) | 0.409 |
| **Myocardial bridge** | 1.474 (1.144, 1.899) | 0.003 | 1.362 (1.026, 1.809) | 0.033 |

| **Table S4. Severity of Each Item of SSD Among Patients with MB** | | | | | |
| --- | --- | --- | --- | --- | --- |
| **Item** | **Normal** | **Abnormal^a^** | **Mild** | **Moderate** | **Severe** |
| **Dizziness, n(%)** | 142 (42.1) | 195 (57.0) | 145 (43.0) | 41 (12.2) | 9 (2.7) |
| **Trouble sleeping, n(%)** | 113 (33.5) | 224 (66.5) | 120 (35.6) | 73 (21.7) | 31 (9.2) |
| **Tired, n(%)** | 112 (33.2) | 225 (66.8) | 134 (39.8) | 77 (22.8) | 14 (4.2) |
| **Losing interest, n(%)** | 163 (48.4) | 174 (51.5) | 111 (32.9) | 46 (13.6) | 17 (5.0) |
| **Chest pain, n(%)** | 45 (13.4) | 292 (86.6) | 151 (44.8) | 116 (34.4) | 25 (7.4) |
| **Anxious, n(%)** | 174 (51.6) | 163 (48.4) | 119 (35.3) | 38 (11.3) | 6 (1.8) |
| **Worried, n(%)** | 178 (52.8) | 159 (47.2) | 112 (33.2) | 42 (12.5) | 5 (1.5) |
| **Reduced attention, n(%)** | 112 (33.2) | 225 (66.8) | 153 (45.4) | 61 (18.1) | 11 (3.3) |
| **Bloating, n(%)** | 143 (42.4) | 194 (57.6) | 136 (40.4) | 51 (15.1) | 7 (2.1) |
| **Muscle pain, n(%)** | 141 (41.8) | 196 (58.2) | 127 (37.7) | 59 (17.5) | 10 (3.0) |
| **Sensitive, n(%)** | 274 (81.3) | 63 (18.7) | 49 (14.5) | 14 (4.2) | 0 (0.0) |
| **Numbness, n(%)** | 161 (47.8) | 176 (52.2) | 118 (35.0) | 48 (14.2) | 10 (3.0) |
| **Blurry vision, n(%)** | 152 (45.1) | 185 (55.0) | 135 (40.1) | 44 (13.1) | 6 (1.8) |
| **Agitated, n(%)** | 203 (60.2) | 134 (40.0) | 100 (29.7) | 32 (9.5) | 2 (0.6) |
| **Obsessive-compulsive, n(%)** | 284 (84.3) | 53 (15.8) | 45 (13.4) | 8 (2.4) | 0 (0.0) |
| **Skin allergies, n(%)** | 202 (59.9) | 135 (40.1) | 101 (30.0) | 32 (9.5) | 2 (0.6) |
| **Health concerns, n(%)** | 172 (51.0) | 165 (48.9) | 129 (38.3) | 29 (8.6) | 7 (2.1) |
| **Difficulty breathing, n(%)** | 182 (54.0) | 155 (46.0) | 113 (33.5) | 38 (11.3) | 4 (1.2) |
| **Choking, n(%)** | 196 (58.2) | 141 (42.0) | 92 (27.3) | 43 (12.8) | 6 (1.8) |
| **Frequent urination, n(%)** | 230 (68.2) | 107 (31.7) | 80 (23.7) | 24 (7.1) | 3 (0.9) |

^a^ "Abnormal" represents the sum of the "Mild," "Moderate," and "Severe" categories.

**Figure S1. The Somatic Symptom Scale-China^[1]^**


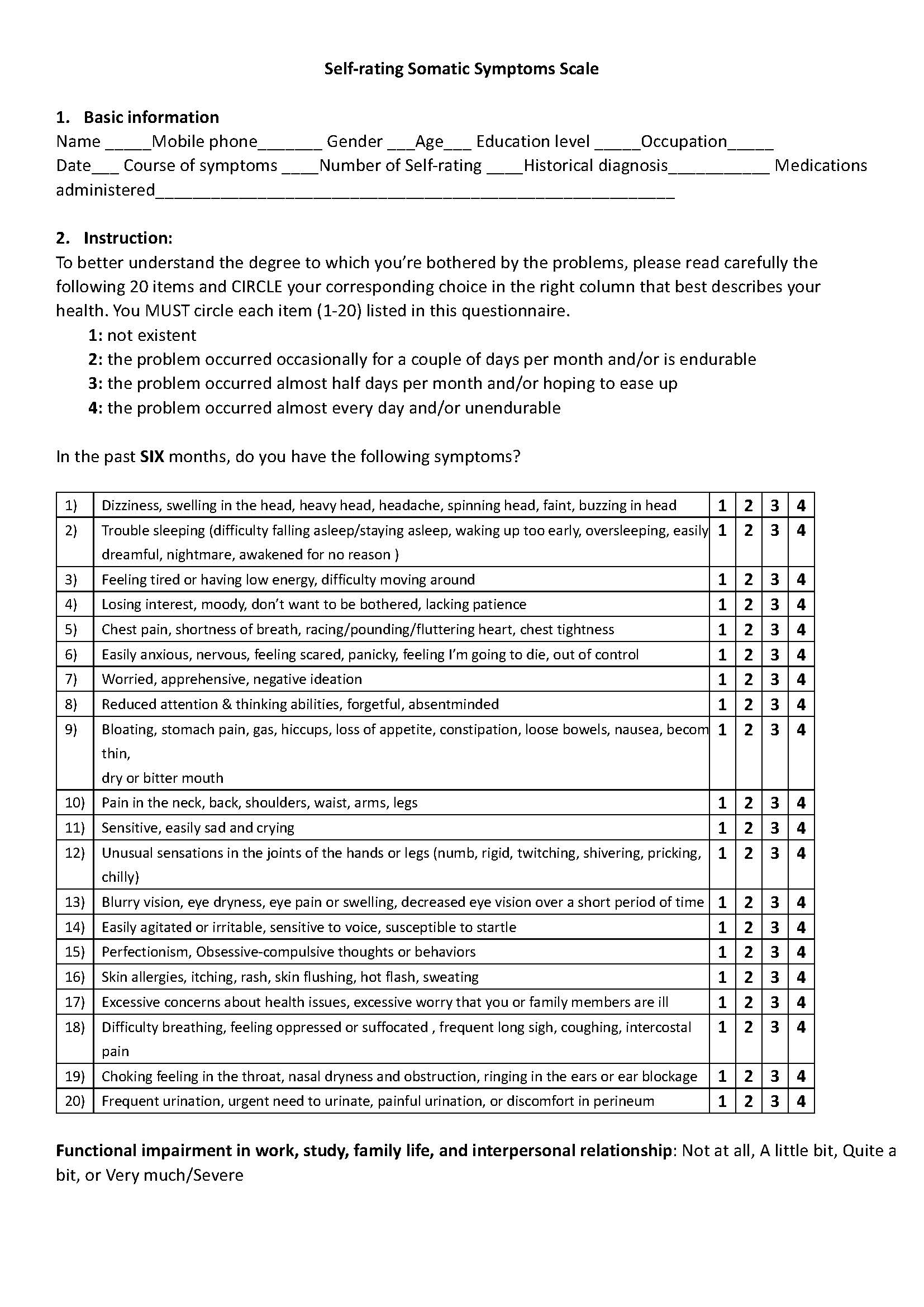


**Figure S2. Flow Chart. SSS-CN, Somatic Symptom Scale-China questionnaire; PHQ-9, Patient Health Questionnaire-9; GAD-7, Generalized Anxiety Disorder-7. Clinical trial number NCT04664387.**


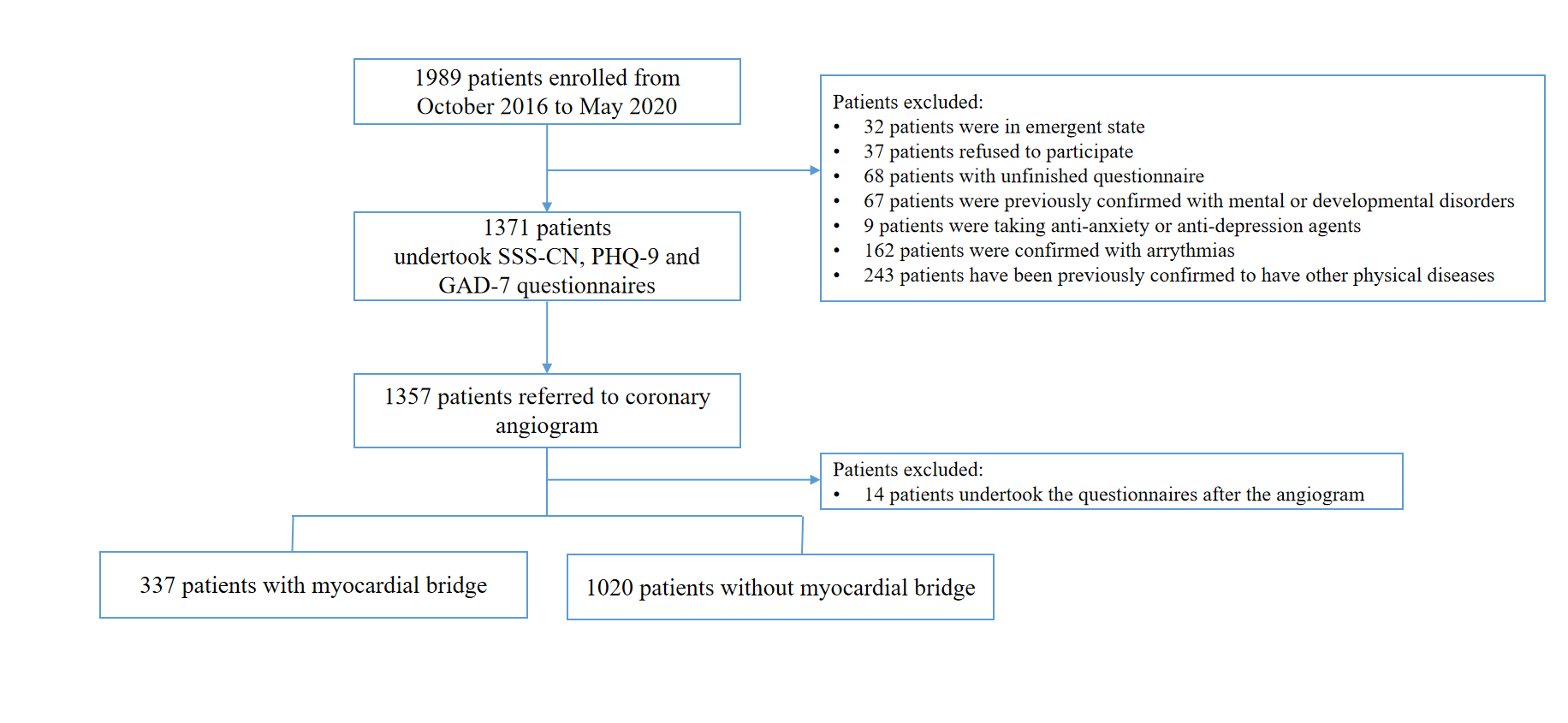


**Reference**

[1] Zhuang Q, Mao JL., Li CB, & He B. Preliminary development of the self⁃rating somatic symptom scale and the study on its reliability and validity. Chinese Journal of behavioral medicine and brain science, 2010, 19:847⁃849.
